# Congenital complete arhinia with alobar holoprosencephaly

**DOI:** 10.4314/gmj.v56i3.14

**Published:** 2022-09

**Authors:** Adwoa P Boakye-Yiadom, Samuel B Nguah, Haruna Mahama, Gyikua Plange-Rhule

**Affiliations:** 1 Directorate of Child Health, Komfo Anokye Teaching Hospital, Kumasi, Ghana; 2 Department of Child Health, Kwame Nkrumah University of Science and Technology, Kumasi

**Keywords:** Arhinia, holoprosencephaly, Respiratory Distress

## Abstract

Congenital arhinia is a life-threatening, rare craniofacial disorder, which, when not identified and managed early can cause severe respiratory distress at birth due to upper airway obstruction. Since neonates are obligate nasal breathers, simultaneous sucking and breathing requirement in neonates with arhinia leads to respiratory distress. Inspiration and expiration through the oral passage alone may result in thoracic retraction, thereby further exacerbating respiratory distress.

We report a rare case of congenital complete arhinia with alobar holoprosencephaly in a 9-month-old. With no family history of congenital malformations, maternal risk factors and uneventful pregnancy, a term female neonate was delivered vaginally without immediate post-delivery respiratory distress. Examination revealed microcephaly, absent fontanelles, fused cranial sutures and bilateral microphthalmia. Breathing was spontaneous, with no immediate signs of respiratory distress. An additional diagnosis of alobar holoprosencephaly was made after a head computed tomography (CT) scan was done. Management included the initial stabilisation phase of supplemental oxygen and an orogastric tube for feeding. The baby did not require both tracheostomy and gastrostomy tubes, as she was not in severe respiratory distress requiring a tracheostomy tube nor having difficulties feeding with the orogastric tube.

## Introduction

Congenital arhinia is the partial or complete absence of the external nose, nasal cavity, and olfactory apparatus at birth, which can occur alone but is generally associated with other midline defects. Arhinia is presumed to result from a specific defect in the nasal placodes or surrounding neural crest-derived tissues during embryonic development.^1^ It is variably associated with absent paranasal sinuses, hypertelorism, microphthalmia, colobomas, nasolacrimal duct abnormalities, midface hypoplasia, high-arched palate, absent olfactory bulbs and defects of the reproductive axis in males.^2^ Arhinia is evident at birth. Since neonates are obligate nasal breathers, respiratory distress from severe upper airway obstruction is usually noted, though not always; prompt airway management of these babies is key to their survival.

It is a rare condition first seen in 1931, with few cases documented in the literature. The pathogenesis is not well understood due to the scarcity of cases; however, there is an association with other craniofacial malformations.^2^ DNA sequencing of 40 people with arhinia showed 84 per cent had a missense mutation in the SMCHD1 gene.^3^

Holoprosencephaly is a developmental disorder of the brain resulting from the defective formation of the prosencephalon and inadequate induction of forebrain structures. The abnormality, which represents a spectrum of severity, is classified into alobar, semi-lobar, and lobar, depending on the cleavage abnormality. Some cases of holoprosencephaly are characterised by various midline craniofacial malformations.^4^

**Figure 1:**
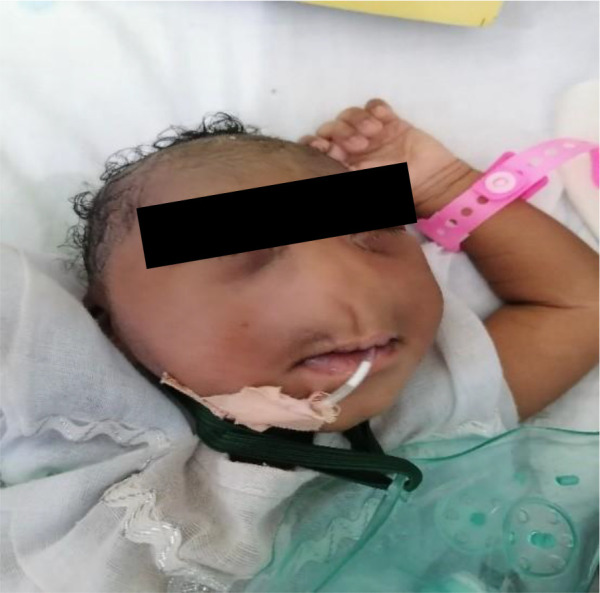
Infant with complete arhinia. Note the flat nasal bridge and complete absence of the external nose.

We describe a rare case of congenital arhinia with alobar holoprosencephaly in a 9-month-old child (Figure 1) after obtaining consent from both parents.

## Case Report

A 39-year-old para 8, cocoa farmer with no underlying chronic illness had her regular antenatal clinic visits at a primary care facility after a planned pregnancy. She had an uneventful pregnancy, attending all scheduled antenatal visits. She underwent routine antenatal tests, including first and third-trimester HIV antibody tests and Venereal Disease Research Laboratory blood test for syphilis. The results of these tests were all negative. She also underwent two prenatal ultrasound scans but not foetal anomaly scans.

Both results indicated a normal developing singleton pregnancy. She was not on long-term medications before and during the pregnancy apart from the routine antenatal vitamins and irons. She had no documented history of gestational diabetes mellitus or hypertension and did not smoke or consume alcohol. Parents are non-consanguineous with no family history of congenital malformations.

Labour lasted less than three hours as the mother delivered a term female baby vaginally at a secondary healthcare facility. The baby cried at birth with suctioning of the mouth, but no active resuscitation was required since she was in a good condition. She weighed 3.0kg and had Apgar scores of 8 and 9 at 1 and 5 minutes, respectively. There was no noticeable birth injury; however, the attending midwife noted the obvious facial deformity and immediately contacted the Komfo Anokye Teaching Hospital (KATH), a tertiary hospital, for onward transfer and further management. The baby was transported in an ambulance with supplemental oxygen delivered via a non-rebreather mask and oxygen saturation within normal limits throughout the transfer period. She was received at KATH and immediately transferred to the neonatal intensive care unit (NICU).

At the NICU, breathing was spontaneous with a respiratory rate of 52 cycles per minute, heart rate of 170 beats per minute, a random blood sugar of 9.4mmol/L, a temperature of 37.2oC and oxygen saturation of 98% on room air. After an hour, an oropharyngeal airway was inserted, and oxygen delivery mode with the non-rebreather mask was maintained as her oxygen saturation dropped from 98% to 85%. An orogastric feeding tube was subsequently passed to prevent aspiration during feeding. Further examination revealed microcephaly (head circumference of 26cm), absent fontanels, fused sutures and bilateral microphthalmia. She, however, had no cleft lip or palate, abdominal wall defects, spinal deformities or ambiguous genitalia.

A multidisciplinary team comprising the ENT surgeons, the paediatric pulmonologist, the paediatric surgeons, the paediatric neurologist, and the maxillofacial surgeons was set up to facilitate care. Input from the team members was expected to include possible tracheostomy insertion to aid in maintaining the airway, gastrostomy tube insertion to prevent aspiration and facilitate feeding and facial reconstructive surgery.

**Figure 2:**
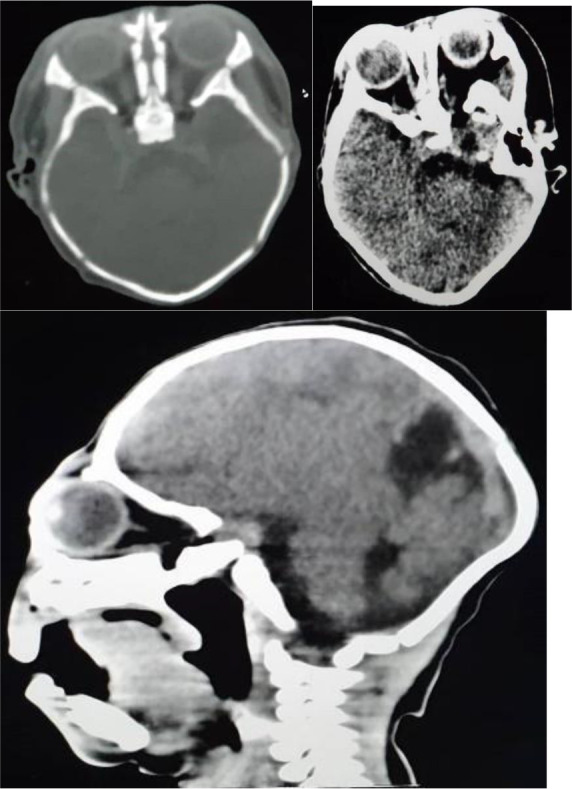
Head CT scan showing posteriorly situated mal-developed mono-ventricle communicating with a dorsal cyst, absent falx, inter-hemispheric fissure and corpus callosum and fusion of the thalami.

The team planned to insert a gastrostomy tube for feeding if difficulty in coordinating feeding arose. A tracheostomy tube was not inserted because the baby could breathe and sustain good oxygen saturation with or without feeding. As the baby had microcephaly and arhinia, the team performed a CT scan to delineate structures in the brain and upper airway (Figure 2).

The CT scan showed a posteriorly situated, mal-developed monoventricle (communicating with a dorsal cyst) with absent falx, inter-hemispheric fissure and corpus callosum. Also noted was a fusion of the Thalami. The structures in the posterior fossae, however, appeared normal. There was also a reduction in the inter-ocular distance consistent with hypotelorism. Other structures appeared normal, thus, suggestive of alobar holoprosencephaly.

**Figure 3:**
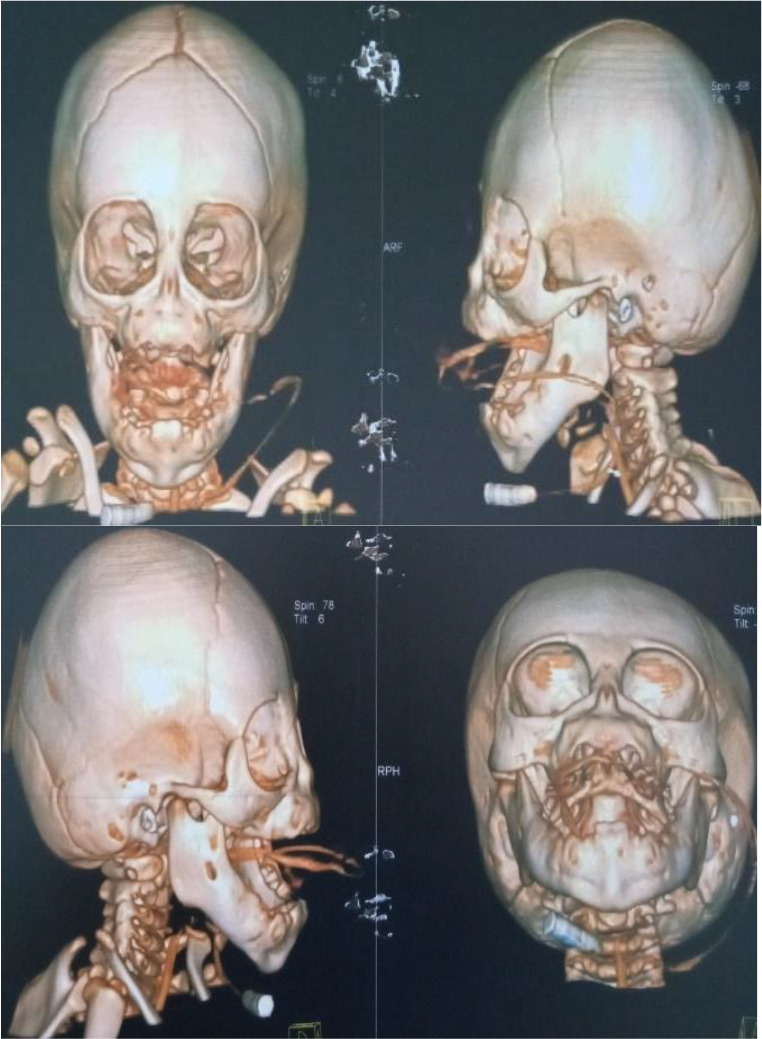
A 3D Head and upper airway CT scan showing complete absence of the nasal bone, internal nasal structures and complete stenosis of the nasal canals.

A 3D CT scan of the head and upper airway confirmed the above findings. They showed a complete absence of the nasal bone and the internal nasal structures with complete stenosis of the nasal canals, as shown in Figure 3 below.

At 9 months old, a child has a significant developmental delay, though not surprising. At her age, she only has good head control, a palmer grasp, turns her head too loud sounds and smiles back. She is currently being seen by a rehabilitation medicine specialist and a paediatric neurologist. She has been weaned off the intranasal oxygen and sustains good oxygen saturations, though she still has a mild sternal recession and tracheal tugging. She is also tolerating feeds by mouth very well with no difficulty. This mode of feeding will be continued till the medical team decides whether a gastrostomy tube be inserted or otherwise. The baby is scheduled to undergo exploratory surgery to observe the nasal structures directly.

## Discussion

The case of a 9-month-old baby born with a very rare condition of complete congenital arhinia is described above. She was born with no immediate respiratory distress at birth (which subsequently developed). She had an initial difficulty feeding by mouth, hence was feeding via an orogastric tube. She, however, has alobar holoprosencephaly in addition to the arhinia, necessitating possible reconstructive facial surgery and multidisciplinary developmental care. Arhinia is a rare and potentially life-threatening condition which is easy to recognise at birth but might be difficult to manage in terms of preventing complications such as aspiration.

Though congenital arhinia is associated with some genetic predisposition, as mentioned above, the anomaly occurs due to some failed processes during the development of the nose.^4^ Most reported cases of congenital arhinia were diagnosed only after delivery, as in our patient, except for five reported cases of isolated arhinia which were detected during the antenatal scan. All the cases were detected in the second trimester, ranging from 23 to 29 weeks.^5^

Prenatal diagnosis of arhinia is important for parents and clinicians to plan the delivery and care afterwards. However, the chances of detecting arhinia during an antenatal scan would largely depend on the skills of ultrasonographers as well as the quality of sonography machines used. It is, therefore, not surprising that this case was completely missed till after delivery, especially in a country where formal foetal anomaly scans are still a rarity.

The main issues associated with congenital arhinia are severe airway obstruction, respiratory distress and inability to feed, though most cases have a normal life.^6^ An oropharyngeal airway, surgically created nasal airway or a tracheostomy tube is an important part of early management. The feeding problems can also be addressed if the airway is secured by any of the measures above or by placing an orogastric tube or a gastrostomy tube.^7^ The management of our patient so far has been based on clinical problems developed. The baby currently has mild respiratory distress (mild sternal recessions and tracheal tugging), though saturating well off supplemental oxygen, has a significant developmental delay (motor and speech) but has no feeding difficulties by mouth.

Surgical reconstruction of arhinia is very complex and should be performed only by a multidisciplinary team of otorhinolaryngologists, plastic surgeons, and prosthodontists. The arhinia reconstruction process mainly consists of 2 parts: reconstruction of the nasal cavity^8^ and reconstruction of the external nose.

This is also achieved with the placement of skin expanders on the forehead, an external nose can then be created with the use of the expanded forehead flap with local perinasal flaps and costochondral grafts.^9^ Surgical reconstruction of the external nose and inner cavities should consider factors such as the characteristics of nasal growth and psychological impact on the child. Reconstruction can be delayed at least until the preschool years, when facial development is nearly completed.^10^

The embryological development of the nose occurs between the third and tenth week of life.^11^ Pathogenesis of arhinia is still not clearly understood. Few postulations of how arhinia may develop include: Failure of medial and lateral nasal processes to grow, premature fusion of the nasal medial processes or lack of resorption of the nasal epithelial plugs.^12^ In the 6th week, the maxillary and frontal process fuses to form the rudimentary palatal shelves. Cells within the nasal pits continue to migrate posteriorly to form the primitive nasal cavities, separated from the buccal cavity by the rudimentary palatal shelves. Merging of the said processes occurs in week 7 and week 8; however, in cases of congenital arhinia, the lateral processes and maxillary processes fail to fuse, which results in the anomaly.^13^

Deformity gene factors and problems from the mother are still unknown. A case reported by Ruprecht et al.^14^, showed that 2 sisters were born into a healthy family and suffered from arhinia at the same time. However, in our case, this is the first of its kind in the nuclear and extended family, though genetic studies have not been done.

Other malformations associated with arhinia include craniofacial anomalies such as the absence of paranasal sinuses, hypo- and hypertelorism, microphthalmia, anophthalmia, and colobomata.^4^ These malformations often turn to determine the immediate and long-term prognosis of the patient. The combination of complete arhinia, bilateral microphthalmia, hypotelorism and alobar holoprosencephaly in our patient complicates her management. In addition to the facial reconstructive surgery cosmetically required in this patient, there is the need to monitor and manage both psychosocial and motor skills as this child grows.

## Conclusion

Congenital arhinia is still a rare condition with few reported cases. Our case is unique because of its association with alobar holoprosencephaly, an association that is not common.

## References

[1] Zhang Mao-mao, Hu Yang-hong, He Wei, Hu Kui-kui (2014). “Congenital arhinia: a rare case.”. The American Journal of Case Reports.

[2] Blair Vilray Papin, Brown James Barrett (1932). “Nasal abnormalities, fancied and real: The reaction of the patient: Their attempted correction.”. International Journal of Orthodontia, Oral Surgery and Radiography.

[3] Gordon Christopher T, Xue Shifeng, Yigit Goekhan, Filali Hicham, Chen Kelan, Rosin Nadine, Yoshiura Kohichiro (2017). “De novo mutations in SMCHD1 cause Bosma arhinia microphthalmia syndrome and abrogate nasal development.”. Nature genetics.

[4] Raam Manu S, Solomon Benjamin D, Muenke Maximilian (2011). “Holoprosencephaly: a guide to diagnosis and clinical management.”. Indian pediatrics.

[5] Leroy D, Slachmuylder E, Popijn M, Cassart M, Massez A, D'Haene N, Désir J (2016). “Antenatal Diagnosis of Isolated Total Arhinia in the Second Trimester of Pregnancy.”. Open Journal of Obstetrics and Gynecology.

[6] Meng L J, Huang Z L, Niu Lei (2009). “Congenital arhinia: one case report.”. Zhonghua er bi yan hou tou Jing wai ke za zhi= Chinese Journal of Otorhinolaryngology Head and Neck Surgery.

[7] Hou Jia-Woei (2004). “Congenital arhinia with de novo reciprocal translocation, t (3; 12)(q13. 2; p11. 2).”. American Journal of Medical Genetics Part A.

[8] George H, Gifford J R, Swanson Lennard, Donald W Maccollum (1972). “Congenital absence of the nose and anterior nasopharynx: Report of two cases.”. Plastic and Reconstructive Surgery.

[9] Brusati Roberto, Colletti Giacomo (2012). “The role of maxillary osteotomy in the treatment of arhinia.”. Journal of Oral and Maxillofacial Surgery.

[10] Mühlbauer Wolfgang, Schmidt Andreas, Fairley Jeffrey (1993). “Simultaneous construction of an internal and external nose in an infant with arhinia.”. Plastic and reconstructive surgery.

[11] Dubourg Christèle, Lazaro Leïla, Pasquier Laurent, Bendavid Claude, Blayau Martine, Le Duff Franck, Durou Marie-Renée, Odent Sylvie, David Véronique (2004). “Molecular screening of SHH, ZIC2, SIX3, and TGIF genes in patients with features of holoprosencephaly spectrum: mutation review and genotype-phenotype correlations.”. Human mutation.

[12] Nishimura Y (1993). “Embryological study of nasal cavity development in human embryos with reference to congenital nostril atresia.”. Cells Tissues Organs.

[13] Lee K J, Farrior J (1991). “Embryology of clefts and pouches.”. Essential Otolaryngology Head and Neck Surgery.

[14] Ruprecht K W, Majewski F (1978). “Familiary arhinia combined with peters' anomaly and maxilliar deformities, a new malformation syndrome (author's transl).”. Klinische Monatsblatter fur Augen-heilkunde.

